# Further Histological and Cellular Characterization of Hidradenitis Suppurativa in 11 Patients

**Published:** 2019-12-03

**Authors:** Saira Nisar, Jeffrey L. Roberson, Bonnie C. Carney, Abdulnaser Alkhalil, Lauren T. Moffatt, Jeffrey W. Shupp

**Affiliations:** ^a^Firefighters’ Burn and Surgical Research Laboratory, MedStar Health Research Institute, Washington, DC; ^b^The Burn Center, MedStar Washington Hospital Center, Washington, DC; ^c^Department of Surgery, The George Washington University School of Medicine and Health Sciences, Washington, DC; ^d^Department of Biochemistry and Molecular and Cellular Biology, Georgetown University School of Medicine, Washington, DC; ^e^Department of Surgery, MedStar Georgetown University Hospital, Washington, DC

**Keywords:** hidradenitis suppurativa, cytoarchitecture, immunohistochemistry, wound healing, chronic inflammation

## Abstract

**Background:** Hidradenitis suppurativa is a chronic inflammatory skin disease, with significant morbidity secondary to its recurrent painful and exudative lesions. Given limited research on the cytoarchitecture of hidradenitis suppurativa, this study describes the microscopic structure and cell surface markers present in hidradenitis suppurativa tissue to better understand the disease and identify potential therapeutic targets. **Methods:** Skin biopsies of hidradenitis suppurativa lesions from patients who underwent surgical excision (n = 11) were compared with grossly normal-appearing perilesional skin (n = 5) and normal skin biopsies from unaffected individuals (n = 4). Histopathology and epidermal thickness were assessed using hematoxylin and eosin and picrosirius red staining, and CD3, a T-cell marker, and CD31 (PECAM), a vascular endothelial cell marker, were assayed using immunofluorescence. Data were analyzed and compared using analysis of variance and Student's *t* test. **Results:** Histological examination showed that hidradenitis suppurativa samples had a significantly thicker epidermal layer than normal skin (335.23 ± 165.01 µm vs 57.24 ± 18.43 µm, *P* = .005), extending into and engulfing the dermis. The hidradenitis suppurativa dermis had extensive cellular infiltration and aggregation as well as disorganized collagen. Immunofluorescence analysis revealed that, at the dermal level, hidradenitis suppurativa lesions had a significantly greater quantity of CD3^+^ (324.29 ± 139.28 vs 14.93 ±16.32, *P* < .0001) and CD31^+^ (322.15 ± 155.46 vs 2.84 ± 5.56, *P* < .0001) cells/mm^2^ compared with normal skin samples. **Conclusions:** Hidradenitis suppurativa lesions have thicker epidermal layers, more dermal cellular infiltrate, and disorganized collagen fibers compared with normal skin. Furthermore, hidradenitis suppurativa dermis has a greater quantity of CD3^+^ and CD31^+^ cells than normal skin.

Hidradenitis suppurativa (HS) is a chronic inflammatory skin disease that classically presents as recurrent painful, exudative lesions, most often found in intertriginous areas with a high density of apocrine glands. While the exact pathophysiology of HS is not known, it is believed to be the result of dysregulation of the local immune system within the sebaceous glands.^1^^,^^2^ Disease prevalence is estimated between 0.05% and 4.10% of the population^1^; however, its morbidity can be significant, related to the debilitating nature of the lesions themselves and the social stigma associated with their purulent and malodorous discharge.^2^

Through perifollicular lymphocytic infiltration, patients progress from isolated nodules (Hurley stage I) to recurrent inflammation and tract formation (Hurley stage II) and, ultimately, to coalesced tracts (Hurley stage III).^2^ Surgical intervention, most often through wide excision and healing by secondary intention, is the mainstay of treatment of advanced HS with fibrosis.^3^ However, adjuvant medical therapy is becoming increasingly more common and surgical treatment is reserved for patients refractory to medical management.^4^

Hypotheses on the etiology of HS have suggested that social factors such as smoking, obesity,^5^^,^^6^ low socioeconomic status,^7^ family history of HS,^8^ and bacterial colonization^9^^,^^10^ are all involved in the development of the lesions. Studies have also investigated the role of cytokines such as IL6, IL10, TNFα, and TGFβ1^11^^-^13 and their products^14^^,^^15^ in the disease course. Small observational studies have demonstrated that early HS lesions are characterized by neutrophilic abscess formation with a histiocytic predominance, whereas chronic lesions experience an expansion of B and plasma cells. Mast cells can be found in all stages of HS skin.^15^

Despite these hypotheses, a complete understanding of the mechanisms of this disease, as well as potential therapeutic targets, remains elusive. Given the paucity of research on the cytoarchitecture and cellular elements common to HS, this study aims to compare HS samples with normal skin (NS) to both corroborate previously reported literature and better understand the disease process. Furthermore, this study aims to identify the specific cellular population present in the dermis of HS with the aim to ultimately identify potential therapeutic targets.

## MATERIALS AND METHODS

### Study design

This retrospective cohort study characterized histology of HS skin and compared it with NS. Hidradenitis suppurativa samples were obtained from 11 patients who underwent surgical resection. Depending on excision size, grossly normal-appearing perilesional skin was assessed when available. Healthy skin controls were taken from a separate patient cohort of nondiseased panniculectomy samples that would otherwise have been discarded. From each sample, a 3-mm punch biopsy was taken for histopathological analysis. Patients’ demographic information was obtained by performing a retrospective medical record review. The MedStar Health Research Institute institutional review board granted approval for this study, and samples and data were anonymized to maintain patient confidentiality.

### Cytoarchitectural analysis

Skin samples were fixed in 10% formalin, embedded in paraffin, and sectioned on a microtome (Leica, Germany) to 6-µm thickness. Slides were dried overnight. Xylene was used to remove paraffin, and alcohol gradient was used to rehydrate slides before staining with hemotoxylin and eosin (H&E) stains. A Zeiss microscope (Carl Zeiss, Oberkochen, Germany) was used to image HS, perilesional skin, and NS slides at 10× magnification for a side-by-side cytoarchitectural analysis.

### Collagen-Specific Staining

To further characterize collagen distribution of the dermis, picrosirius red stain and polarized light microscopy were used as described previously.^16^ First, the slides were de-waxed using xylene and hydrated using alcohol gradient, followed by staining with hematoxylin. Subsequently, slides were stained with picrosirius red and washed using acidified water, followed by dehydration with ethanol and xylene. Zeiss microscope (Carl Zeiss) was then used to image HS, perilesional skin, and NS slides at 10× magnification using polarized light microscopy.

### Assessment of epidermal thickness

ImageJ (v1.52a; NIH, Bethesda, Md) software was used to assess and compare the epidermal thickness of HS lesions, perilesional skin, and NS. Three measurements were taken from the top of the epidermis to the dermal-epidermal junction at the widest and narrowest parts of HS, perilesional skin, and NS (see Supplementary Figure 1, available at: www.eplasty.com). These thicknesses were averaged and analyzed using one-way analysis of variance with multiple comparisons and Tukey's correction with significance value of *P* < .05 on GraphPad Prism 6.0 (La Jolla, Calif).

### Immunofluorescence analysis

To investigate the nature of cellularity of the samples, immunofluorescence (IF) was conducted in HS (n = 11) and NS samples (n = 4). All samples were stained with anti-CD3 (abcam ab5690) and anti-CD31 (abcam ab24590) antibodies and visualized under IF. A standard staining and antigen retrieval procedure was utilized with anti-CD3 and anti-CD31 solutions at a 1:100 concentration. Negative staining controls were incorporated by replacing the primary antibody of interest with antibody diluent. Six regions of interest (3 epidermal and 3 dermal) were selected per sample, and cellular quantification was conducted at 40× magnification using Zeiss microscope (Carl Zeiss). The quantity of both cell types at the epidermal and dermal levels was compared with Student's *t* test at a significance level of *P* < .05 using GraphPad Prism 6.0.

## RESULTS

### Baseline characteristics

Lesional skin biopsies were collected from 11 patients who underwent surgical excision of HS. Grossly normal-appearing perilesional skin could be obtained from 5 of these patients. All 11 patients were African American. Fifty-five percent (n = 6) of patients were male with an average age of 37 ± 12 years. Mean body mass index was 36.27 ± 13.53 kg/m^2^, and 55% (6) of individuals were active smokers. Hurley stage III disease with coalesced tracts was present in 82% (n = 9) of the cohort. Oral antibiotics were attempted in 27% (3); 91% (10) had undergone prior incision and drainage procedures, and 27% (3) had prior operative intervention (Table 1).

#### Cytoarchitecture

##### Epidermis

**Figure 1:**
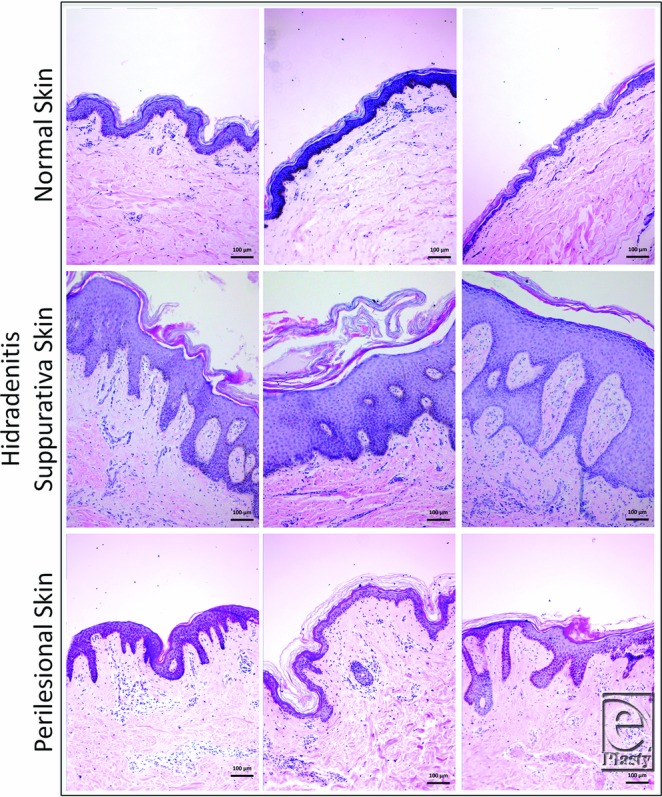
Thicker epidermis and increased irregularity of rete ridges in HS compared with perilesional and normal skin (H&E stain). HS epidermis extension into the dermis results in dermal islands. HS indicates hidradenitis suppurativa. Scale bar = 100 µm, 10× magnification.

Histopathological analysis showed that HS samples contained thicker epidermal layers than perilesional skin and NS, with increased frequency and irregularity of rete ridges. Furthermore, HS epidermis extended into and engulfed the dermis, resulting in dermal islands within the epidermis in 8 of 11 samples. Specifically comparing NS and perilesional skin histomorphology, perilesional samples demonstrated similar epidermis and rete ridges appearance (Fig 1). Histological slides from all 11 samples are shown in Supplementary Figure 2 (available at: www.eplasty.com).

**Figure 2:**
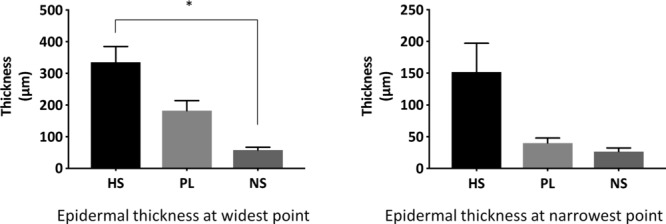
Comparison of epidermal thickness in HS skin versus PL and NS samples. Epidermal thickness at the widest point: HS versus NS, *P* < .05, HS versus perilesional skin, and perilesional versus NS, *P* > .05. Epidermal thickness at the narrowest point: not significant. HS indicates hidradenitis suppurativa; PL, perilesional; and NS, normal skin. * represents statistically significant values i.e., *P* < .05.

At the widest portion, epidermal thickness was significantly greater in HS samples than in NS samples (335.23 ± 165.01 µm vs 57.24 ± 18.43 µm, *P* = .005). However, there was no significant difference in thickness between HS and perilesional skin (335.23 ± 165.01 µm vs 182.12 ± 71.38 µm, *P* = .107) or between NS and perilesional skin (57.24 ± 18.43 µm vs 182.12 ± 71.38 µm, *P* = .355). Furthermore, the difference at the narrowest section of epidermis was not significant between HS and NS (151.74 ± 150.62 µm vs 26.47 ± 11.22 µm, *P* = .183), between HS and perilesional skin (151.74 ± 150.62 µm vs 40.16 ± 16.99 µm, *P* = .204), or between perilesional skin and NS (40.16 ± 16.99 µm vs 26.47 ± 11.22 µm, *P* = .983) (Fig 2).

##### Dermis

**Figure 3:**
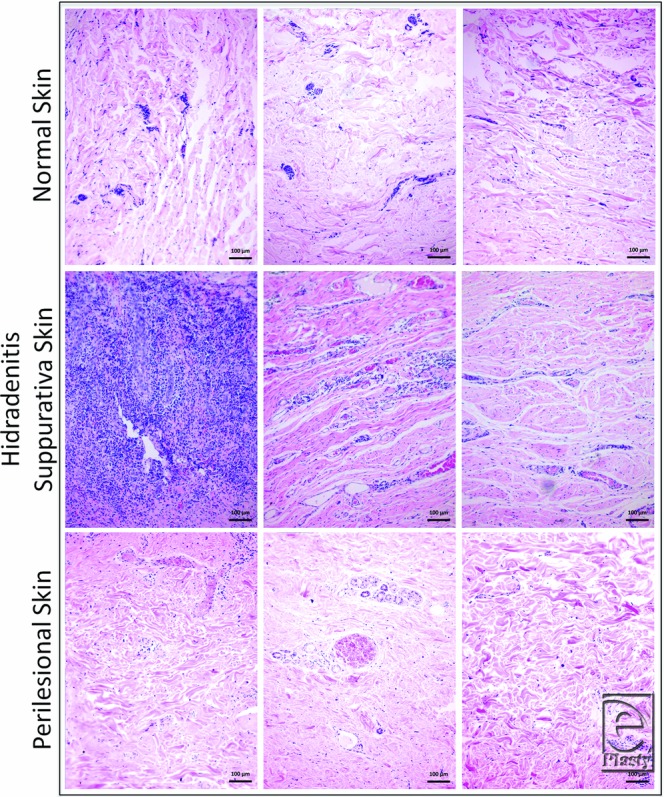
Cellular infiltration in hidradenitis suppurativa dermis compared with perilesional and normal skin (H&E stain). Scale bar = 100 mm, 10× magnification.

**Figure 4:**
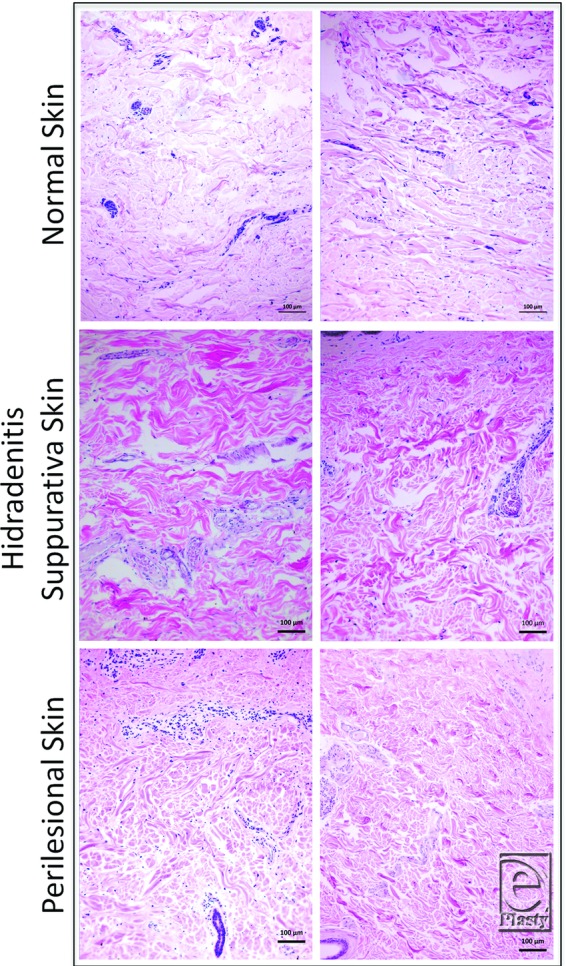
Disorganized collagen fibers in hidradenitis suppurativa dermis compared with perilesional and normal skin (H&E stain). Scale bar = 100 µm, 10× magnification.

**Figure 5:**
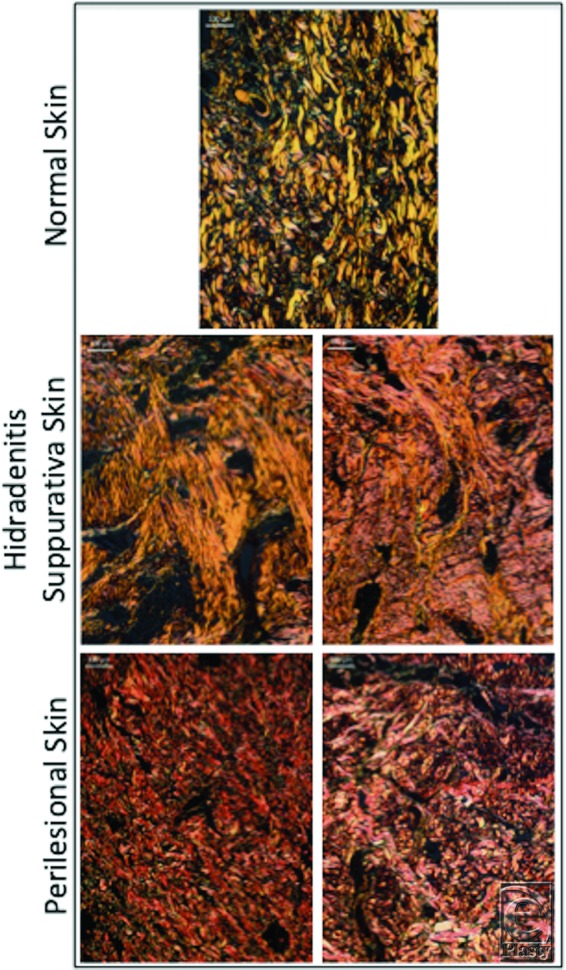
Collagen-specific staining shows both type I (yellow) and III (green) collagen in normal and perilesional skin dermis, compared with predominance of type I collagen (yellow) in hidradenitis suppurativa dermis (picrosirius red stain). Scale bar = 100 µm, 10× magnification.

**Figure 6:**
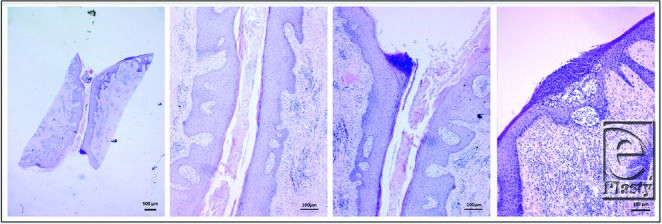
Epithelialized tract in hidradenitis suppurativa sample (H&E stain). 1.25× magnification showing the length of the tract (left). 10× magnification of the tract showing epithelialized tissue (middle). Surface of an abscess (right).

There was extensive cellular infiltration in 91% (10) of HS samples compared with all healthy skin where little to no infiltration was observed (Fig 3). In 9% (n = 1) of HS samples, infiltration was present around the hair follicle. Collagen fibers were arranged in a disorganized or random fashion in the dermis of all HS specimens compared with perilesional skin and NS (Fig 4). Collagen-specific staining revealed the presence of collagen types I and III in normal and perilesional skin dermis; however, HS dermis contained predominantly type I collagen (Fig 5). An epithelialized tract with cellular infiltration was seen within the dermis of one HS specimen (Fig 6).

### Immunofluorescence analysis

**Figure 7:**
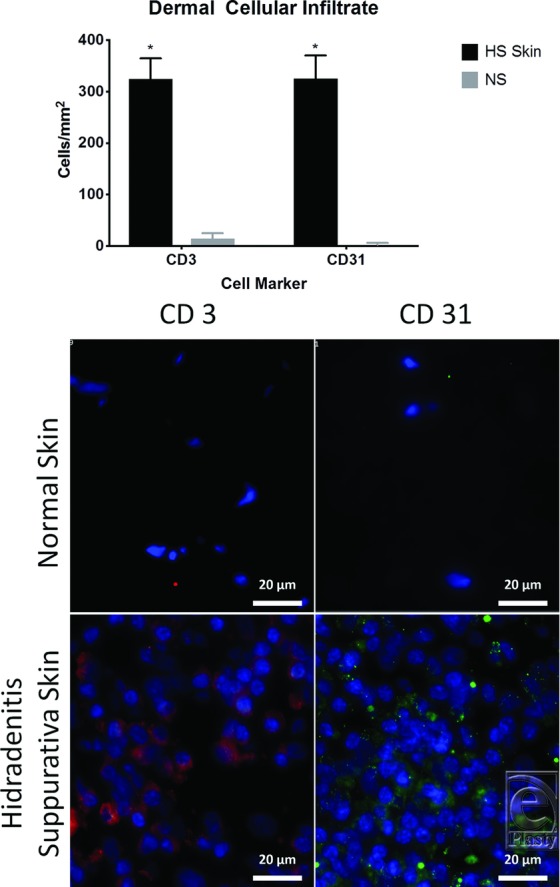
Comparison of dermal CD3 and CD31 infiltration in the dermis of HS skin versus NS using immunofluorescence. (Top) NS; (bottom) HS. Red (CD3), green (CD31), blue (DAPI). *P* < .0001. Scale bar = 20 µm. HS indicates hidradenitis suppurativa; NS, normal skin. * represents statistically significant values i.e., *P* < .05.

Immunofluorescence analysis revealed that, at the dermal level, HS lesions had a significantly greater quantity of CD3^+^ (324.29 ± 139.28 vs 14.93 ± 16.32, *P* < .0001) and CD31^+^ (322.15 ± 155.46 vs 2.84 ± 5.56, *P* < .0001) cells/mm^2^ than NS samples. However, HS lesions had a similar quantity of CD3^+^ (2.28 ± 5.46 vs 6.40 ± 10.37, *P* = .2123) and CD31^+^ (4.27 ± 7.72 vs 2.84 ± 4.20, *P* = .5806) cells/mm^2^ compared with NS samples at the epidermal level (Fig 7).

## DISCUSSION

Hidradenitis suppurativa is a chronic inflammatory skin condition that presents as recurrent painful, draining abscesses, which undergo cycles of inflammation and healing with fibrosis. This condition significantly impacts the daily routine and quality of life of patients, given its relapsing and remitting nature.^1^ Given limited understanding of disease etiology,^17^ refractory cases require a combination of medical and surgical treatment.

### Patient demographics

Surgical management of HS is the last resort for advanced disease that is resistant to medical therapy and drainage,^3^ supported by 82% of patients being Hurley stage III and 91% having undergone previous incision and drainage in this cohort. Furthermore, previously reported risk factors such as smoking and obesity^1^ were present in these patients. Despite family history being implicated in one third of HS cases,^1^ none of the study patients reported other family members with the disease, suggesting that these patients represent the sporadic variety of HS as opposed to the autosomal dominant and familial variant.^8^

### Cytoarchitecture

As compared with NS, HS skin has thicker epidermis, with irregular rete ridges extending into the dermis. In addition, as a result of extension into and engulfment of the dermis, there appears to be dermal islands in the epidermis of HS skin. These findings corroborate most of the previously described HS histology. The similar histological appearance and epidermal thickness of perilesional skin compared with NS could be due to subclinical inflammation. The exact role of these findings in HS development is not clear and could result from recurrent inflammation and lymphocyte infiltration.

Consistent with previous studies,^18^ HS dermis showed extensive lymphocytic infiltration compared with healthy skin, as would be expected during an active inflammatory disease process. Interestingly, the collagen fibers are disorganized and appear distorted in HS skin compared with NS and perilesional skin. Collagen-specific staining revealed HS dermis to contain predominantly type I collagen compared with seemingly equal type I and III collagen in NS and perilesional skin dermis. Repeated cycles of inflammation and healing likely contributed to this disruption of normal cytoarchitecture and increased type I collagen presence. One HS sample contained an epithelialized tract surrounded by lymphocytic infiltration, similar to past reports and consistent with Hurley stage III of the disease.^1^

### Immunohistochemical analysis

The finding of increased quantity of CD3^+^ cells in HS dermis corroborates previous studies, suggesting that the included HS samples are representative of the current understanding of the natural history of the disease.^15^^,^^19^ Although not suggestive of an exact etiology, this finding supports the notion that HS has a profound inflammatory component^20^ and highlights the need for ongoing investigation of the use of anti-inflammatory medications in hopes of preventing progression to tract formation and fibrosis.^21^^,^^22^

Previously, CD31^+^ cells have been shown to be present in the setting of chronic lymphedema in HS.^23^ However, previous studies have not investigated the role of CD31^+^ cells in the setting of HS without chronic lymphedema. CD31, also referred to as platelet endothelial cell adhesion molecule-1 (PECAM-1), is known to be an integral regulator of the inflammatory response, serving as a cell adhesion and signaling receptor expressed on both hematopoietic and endothelial cells. Acute inflammation is associated with elevated CD31 levels, with its main proinflammatory property being facilitation of transendothelial migration of leukocytes.^24^ However, CD31 also has anti-inflammatory effects by suppressing inflammatory cytokine production and maintaining the integrity of the vascular barrier.^24^ Therefore, future treatment targets could include downregulation of CD31’s proinflammatory nature or encouragement of its anti-inflammatory properties.

Recently, CD31 has been suggested as providing immune privilege to the vascular endothelium.^25^ Furthermore, in the setting of recovery from myocardial infarction, CD31 can be utilized as a marker for endothelial progenitor cells (EPCs) and capillary density.^26^ Specifically in HS, previous studies have suggested a mixed vascularization morphology within active lesions related to the presence of a chronic inflammatory stimulus.^27^ Therefore, the significantly increased levels of CD31^+^ cells in the HS samples suggest that neovascularization is involved in the HS disease course. Further investigation into capillary density and EPCs in HS could elucidate the role of immune privilege and vascular patterns in this disease.

## LIMITATIONS

An inherent weakness of this study is the small sample size, though this is characteristic of other HS clinical studies.^10^^,^^19^^,^^20^ In addition, the retrospective design makes NS unavailable from the surgical patients. Therefore, as panniculectomy specimens were used as NS controls, it was impossible to match the HS samples to NS counterparts. However, the 4 NS samples that were used as controls are from a large repository of NS samples collected from plastic surgery cases over the last 5 years and are representative of the cytoarchitecture that has been observed in many of these samples.

While epidermal thickness varies at different anatomical locations, abdominal panniculectomy skin biopsy samples are comparable controls. Previous studies have reported average epidermal thickness to be 61.3 µm on the abdomen, 55.6 µm on the upper back, 58.2 µm on the inner-upper arm,^28^ and 96.5 µm on the buttock.^29^ Fifty-five percent of the surgical specimens in this study were obtained from the axilla. The average epidermal thickness of the panniculectomy skin in this study was 57.24 ± 18.43 µm, similar to the standard for inner-upper arm. Furthermore, grossly normal-appearing perilesional skin could not be obtained from all samples, given the original surgical specimen size.

To achieve improved visualization and cellular quantification, co-staining for CD3 and CD31 was not conducted. Therefore, cells could not be labeled as dual-positive for CD3/CD31.

To continue to better characterize the etiology and disease process of HS, a prospective study is currently being designed. This study will involve analyzing HS and NS samples from the same patients to characterize histological features as well as molecular signaling pathways to compare variable degrees of gene regulation. These data can provide information on potential therapeutic targets in an effort to avoid extensive surgical intervention and its inherent risks.

## CONCLUSIONS

This study corroborates previously reported literature by demonstrating that the cytoarchitecture of HS lesions is different from that of perilesional skin and NS. Hidradenitis suppurativa lesions have thicker epidermal layers as well as increased dermal cellular infiltrate compared with perilesional skin and NS. Furthermore, characterization of the dermal infiltrate reveals that HS lesions have a preponderance of CD3^+^ and CD31^+^ cells compared with NS, suggesting a role of chronic inflammation and abnormalities in vascularization. Work is ongoing to characterize the involvement of the molecular signaling pathway to identify potential therapeutic targets aside from surgical excision, especially in the sporadic, nonfamilial HS population.

## Figures and Tables

**Table 1:** Baseline characteristics of 11 patients

Characteristics	Mean ± SD (range) or % (n)
Male	55 (6)
Ethnicity—African American	100 (11)
Age, y	37 ± 12 (22-54)
Body mass index, kg/m^2^	36.26 ± 13.53 (20-68)
Smokers	55 (6)
Diabetes mellitus	18 (2)
Clinical stage	
Stage I	9 (1)
Stage II	9 (1)
Stage III	82 (9)
Excision sites	
Axilla	55 (6)
Groin	18 (2)
Perineum	9 (1)
Buttock	9 (1)
Foot	9 (1)
Past medical treatment	
Failed oral antibiotics	27 (3)
Ongoing oral antibiotics	27 (3)
Prior procedural treatment	
Incision and drainage	91 (10)
Operative intervention	27 (3)
